# Targeting of prion-infected lymphoid cells to the central nervous system accelerates prion infection

**DOI:** 10.1186/1742-2094-9-58

**Published:** 2012-03-21

**Authors:** Yael Friedman-Levi, Romana Hoftberger, Herbert Budka, Tehila Mayer-Sonnenfeld, Oded Abramsky, Haim Ovadia, Ruth Gabizon

**Affiliations:** 1 Department of Neurology, The Agnes Ginges Center for Human Neurogenetics, Hadassah University Hospital, Jerusalem, Israel; 2 Institute of Neurology, Medical University of Vienna, Vienna, Austria

**Keywords:** Brain, EAE, immune cells, infiltrates, prion, PrPSc, spinal cord

## Abstract

**Background:**

Prions, composed of a misfolded protein designated PrP^Sc^, are infectious agents causing fatal neurodegenerative diseases. We have shown previously that, following induction of experimental autoimmune encephalomyelitis, prion-infected mice succumb to disease significantly earlier than controls, concomitant with the deposition of PrP^Sc ^aggregates in inflamed white matter areas. In the present work, we asked whether prion disease acceleration by experimental autoimmune encephalomyelitis results from infiltration of viable prion-infected immune cells into the central nervous system.

**Methods:**

C57Bl/6 J mice underwent intraperitoneal inoculation with scrapie brain homogenates and were later induced with experimental autoimmune encephalomyelitis by inoculation of MOG_35-55 _in complete Freund's adjuvant supplemented with pertussis toxin. Spleen and lymph node cells from the co-induced animals were reactivated and subsequently injected into naïve mice as viable cells or as cell homogenates. Control groups were infected with viable and homogenized scrapie immune cells only with complete Freund's adjuvant. Prion disease incubation times as well as levels and sites of PrP^Sc ^deposition were next evaluated.

**Results:**

We first show that acceleration of prion disease by experimental autoimmune encephalomyelitis requires the presence of high levels of spleen PrP^Sc^. Next, we present evidence that mice infected with activated prion-experimental autoimmune encephalomyelitis viable cells succumb to prion disease considerably faster than do mice infected with equivalent cell extracts or other controls, concomitant with the deposition of PrP^Sc ^aggregates in white matter areas in brains and spinal cords.

**Conclusions:**

Our results indicate that inflammatory targeting of viable prion-infected immune cells to the central nervous system accelerates prion disease propagation. We also show that in the absence of such targeting it is the load of PrP^Sc ^in the inoculum that determines the infectivity titers for subsequent transmissions. Both of these conclusions have important clinical implications as related to the risk of prion disease contamination of blood products.

## Background

Prion diseases are a group of fatal neurodegenerative disorders that include Creutzfeldt-Jakob disease and kuru in humans, bovine spongiform encephalopathy in cattle, scrapie in sheep and goats, and chronic wasting disease in deer [1]. This group of diseases is caused by the accumulation of a misfolded and oxidized isoform of PrP^Sc^, a normal membrane protein believed to play a role in the protection against oxidative insults [2,3]. All forms of prion diseases are characterized by long incubation periods, which in humans can sometimes amount to decades [3-6]. Although the exact mechanism of prion propagation is unknown, a general sequence of events has been outlined that is consistent with most of the available data [4]. First, and regardless of the route of infection, prions replicate in lymphoid organs, as shown by the fact that both infectivity and accumulation of PrP^Sc ^are initially detected in the spleens of the infected animals [5-8]. Prions invade the nervous system by a mechanism believed to involve transmigration of infected lymphoid cells as well as retrograde transport in ascending peripheral neural tracts [4,9-12]. Once prions enter the brain, prion replication and PrP^Sc ^accumulation seems to occur faster than in the lymphoid system, leading to the certain death of the affected individuals [13-15]. In the presence of inflammatory conditions affecting peripheral organs, activated lymphoreticular cells induce deposition of PrP^Sc ^and prion infectivity in the sites of infiltration in prion-infected animals [16]. For example, mastitis in sheep results in deposition of PrP^Sc ^in the inflamed mammary glands [17].

While no differences in prion disease incubation time or clinical symptoms were reported in these peripheral infection cases, mice incubating scrapie and induced for experimental autoimmune encephalomyelitis (EAE), an autoimmune inflammatory disease of the central nervous system (CNS) [18,19], died from a progressive neurological disease long before the control mice succumbed to classical scrapie [20]. Surprisingly, mice affected by the scrapie-EAE syndrome showed almost undetectable to high levels of brain PrP^Sc^, demonstrating that there is no correlation between the clinical status of the affected scrapie-EAE mouse and PrP^Sc ^accumulation. Immunostaining studies revealed PrP^Sc ^depositions in the demyelinated white matter spinal cord areas of the co-induced mice, mostly co-localized with hematopoietic cell infiltrates. In classical scrapie, PrP^Sc ^aggregates are mostly found in the gray matter [21].

In this project we investigated the mechanism of prion disease acceleration by CNS inflammation. In particular, we asked whether accelerated prion disease in the co-induced animals requires the targeted delivery of PrP^Sc ^to the CNS by activated immune cells. To this aim, we compared the effect of EAE induction on prion disease kinetics at several time points before and after infection with scrapie and found that acceleration of disease is the strongest at one month after prion infection, when PrP^Sc ^is largely accumulated in immune organs such as the spleen [22]. Next, we inoculated naïve mice with viable or homogenized activated immune cells collected from scrapie-EAE mice and controls. Only the activated viable cells generated CNS inflammation and accelerated prion disease, as compared with non-specific immune activation, or with scrapie brain homogenates with similar PrP^Sc ^loads. Our results therefore suggest that EAE-dependent acceleration of fatal prion disease results from the infiltration of PrP^Sc ^loaded immune cells into the CNS. Additional results presented here indicate that, in the absence of such infiltration, incubation times of prion disease relate mostly to the levels of the inoculated PrP^Sc^.

## Methods

### Animals

Female C57Bl/6 J mice were purchased from Harlan (Hebrew University, Jerusalem, Israel) and housed in the animal care facility in compliance with the standard guidelines for animal care. All experiments were approved by the Institutional Animal Care and Use Committee.

### Prion infection

Four- to five-week-old mice were inoculated by intraperitoneal injection with 100 μL or intracerebral injection with 50 μL of 1% scrapie brain homogenate. Mice were followed closely for disease signs until disease manifestation and killed when too sick to reach food and water.

### Experimental autoimmune encephalomyelitis induction

Five- to eight-week-old female C57BL/6 J mice were immunized with an emulsion containing 300 μg of MOG_35-55 _(70% purified; synthesized at the Hebrew University, Jerusalem, Israel) solubilized in saline and an equal volume of complete Freund's adjuvant (CFA; Sigma, Rehovot, Israel) supplemented with 5 mg/mL of heat-killed mycobacteria *Tuberculosis *H37RA (Difco Laboratories, Detroit, MI, USA). The inoculum (0.2 mL) was injected subcutaneously in both flanks. On the day of inoculation and 48 hours later, 100 ng of pertussis toxin (List Biological Laboratories, Inc. Cambell, CA, USA) in 0.1 mL saline was also administered by intraperitoneal injection. Control groups were inoculated the same way but without MOG_35-55_.

### Passive transfer of experimental autoimmune encephalomyelitis

Nine days post induction mice were killed and cells were obtained from the spleen and lymph nodes. Cells were suspended in Roswell Park Memorial Institute medium (Biological Industries, Beit Haemek, Israel) supplemented with 10% fetal calf serum, 1 mM L-glutamine, antibiotics, MOG_35-55 _(20 μg/mL) and mouse recombinant IL-2 (50 units/mL; Peprotech, Rocky Hill, NJ, USA) and incubated for three days in a CO_2 _humidified incubator. Cells were harvested and live cells were separated with Ficoll-Paque (GE Healthcare Bio-Sciences AB, Uppsala, Sweden) and injected into naïve mice. In control groups of cells extracts, cells were frozen after separation, thawed three times and then injected into naïve mice. On the day of inoculation and 48 hours later, 100 ng of pertussis toxin in 0.1 mL saline was also administered by intraperitoneal injection. Mice were followed for clinical symptoms of EAE and evaluated according to a 0 to 6 point score (0 represents no clinical signs and 6 represents death as a result of disease).

### Western blot: PrP immunoblots of brains and spleens

Brains and spleens from mice in all experimental groups were homogenized in 10 volumes of 10 mM Tris-HCl (pH 7.5) containing 300 mM sucrose. Homogenates were normalized for protein level, digested with 40 μg/mL proteinase K (Sigma), and immunoblotted with anti-PrP monoclonal antibody IPC1 (Sigma) or monoclonal antibody 6H4 (Prionics, Schlieren, Switzerland), 40 μL in each lane for brains and 100 μL in each lane for spleen homogenates.

### Pathological examinations

Histological evaluations were performed on paraffin-embedded sections of brain and spinal cord samples. Sections were stained with luxol fast blue/Periodic acid Schiff (PAS) to assess demyelination. Consecutive sections were used for immunohistochemistry with antibodies against the following targets: macrophages and activated microglia (anti-MAC3; BD Pharmingen, San Diego, CA, USA); T-cells (anti-CD3; Serotec, Oxford, UK); and prion protein (anti-PrP 6H4; Prionics).

### Statistical analysis

The clinical scores of disease in groups of animals with EAE and EAE + scrapie were compared using Kruskal-Wallis one-way analysis of variance on ranks. The survival curves were compared using chi-squared test.

## Results and discussion

### Acceleration of prion disease by experimental autoimmune encephalomyelitis may depend on spleen PrP^Sc ^levels

**Figure 1:**
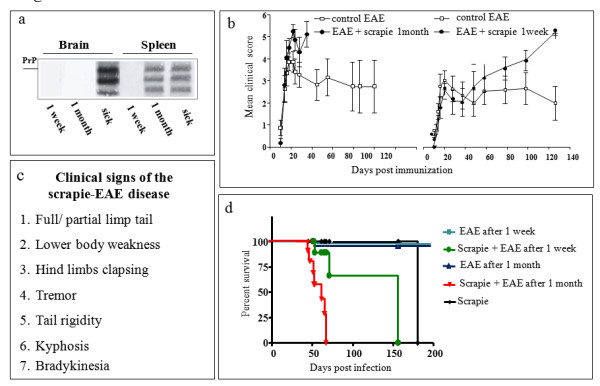
**PrP^Sc ^in spleen is required for the acceleration of prion disease by experimental autoimmune encephalomyelitis**. **(A) **Brain and spleen samples from scrapie-infected mice (C57Bl/6 J, RML strain) at several incubation points (1 week, 1 month, sick) were tested for the levels of proteinase K digested PrP^Sc^. **(B) **Prion-infected mice were induced for MOG-EAE one week (n = 5) and one month (n = 6) after prion infection and followed for signs of disease. The clinical scores of the group induced with EAE one month post infection were significantly different (*P *= 0.025) as compared to naïve mice induced for EAE (n = 6). The clinical scores of mice with EAE induced one week post infection were not statistically different (*P *= 0.5) as compared to naïve mice induced for EAE (n = 6). **(C) **Table presenting chronological symptoms of the scrapie-EAE syndrome. **(D) **Survival curves of mice from the designated groups. Scrapie/EAE at one month was statistically different (*P *= 0.0033) as compared to the corresponding scrapie/EAE at one week.

Regardless of the route of prion infection, PrP^Sc ^is first accumulated in immune organs such as the spleen and lymph nodes and appears in the infected brains much later in the incubation period [22,23]. In the scrapie RML model (mouse), it takes one month after intracerebral or intraperitoneal infection for PrP^Sc ^to accumulate in the spleen at high levels. Indeed, Figure 1A shows that while no PrP^Sc ^was detected in the brain one month after intraperitoneal infection with a scrapie brain homogenate, PrP^Sc ^was easily detectable in the spleen at this time point. To test whether the presence of PrP^Sc ^in immune organs is required for disease acceleration by CNS inflammation, we induced MOG-EAE both one week and one month after scrapie infection (intracerebral injection) of C57Bl/6 J female mice. Animals were followed for clinical symptoms of both EAE and scrapie (Figure 1C). Figure 1B shows no difference in the severity of EAE signs, such as paralysis of the lower limbs, during the first 30 days in the groups induced for EAE after prion infection as compared to control EAE groups [24]. While most mice recovered from the acute disease at this point, the group induced for EAE one month after scrapie infection continued to deteriorate, demonstrating new neurological signs such as tail rigidity, kyphosis, ataxia, tremor and bradykinesia, which are classical characteristics of prion disease [25]. Similar signs were observed at 80 days or later in the group induced for EAE one week after scrapie infection. Figure 1D depicts the survival of mice from these groups as compared to control scrapie-infected mice. Indeed, while mice induced for EAE one week after prion infection showed some acceleration of disease progression (median: 155 days) as compared to scrapie controls (median: 175 days), a much larger difference in survival was observed between the scrapie control (median: 175 days) and the group also induced for EAE at one month of prion infection (median: 60 days). Only a marginal acceleration of disease was observed when EAE was induced in the mice before scrapie infection (not shown). These results indicate that the rate of prion disease acceleration by CNS inflammation may depend on the levels of PrP^Sc ^accumulating in lymphoid organs at the different time points.

### Accelerated disease in mice infected with scrapie-experimental autoimmune encephalomyelitis-activated immune cells

**Figure 2:**
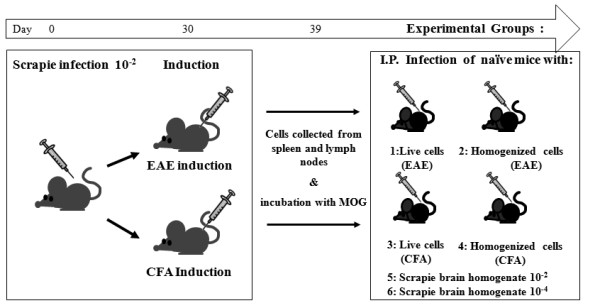
**Outline of scrapie-experimental autoimmune encephalomyelitis transfer experiments**. C57Bl/6 J mice were induced for either MOG-EAE or CFA disease one month after intraperitoneal infection with scrapie RML. Nine days later mice were killed and immune cells collected and incubated with MOG for three days, before their inoculation as viable or homogenate samples into naïve mice. Control scrapie-infected brain samples were also inoculated in naïve mice.

**Figure 3:**
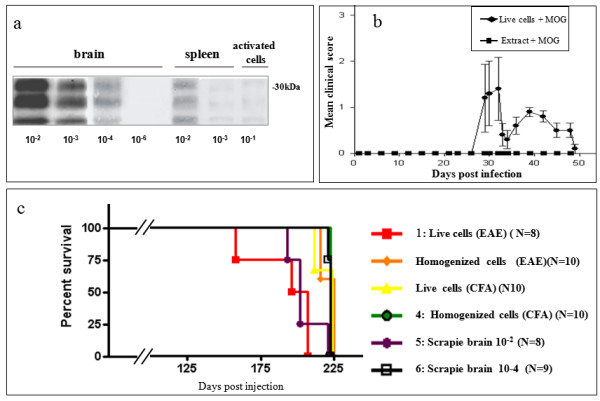
**Infection of mice with viable scrapie-experimental autoimmune encephalomyelitis immune cells shortened the prion disease incubation period**. **(A) **PrP^Sc ^levels in brains and immune samples. **(B) **EAE clinical scores in mice inoculated with viable or homogenated cells after activation *ex vivo *for three days with MOG. **(C) **Survival curves of mice infected with the designated inocula. The survival curve of group 1 was significantly different (*P *< 0.05) as compared to survival curves of groups 2,3,4 and 6. Results represent eight to ten mice in each category.

We next tested the incubation time resulting from prion infection of naïve mice either with viable or with homogenized scrapie-EAE immune cells. To this effect, C57Bl/6 J mice were infected intraperitoneally with scrapie RML prions and one month later induced either for EAE (MOG (myelin oligodendrocyte glycoprotein) + CFA + pertussis toxin) or for adjuvant only inflammation (CFA + pertussis toxin). Nine days thereafter, scrapie-EAE mice and controls were killed, their spleens and lymph nodes harvested and reactivated *in vitro *with MOG and subsequently enriched for viable cells only (see Methods). An outline for these experiments is depicted in Figure 2. Before inoculation into naïve mice groups, cell fractions were tested for PrP^Sc ^levels as compared to sequential dilutions of prion-infected brain and spleen samples (Figure 3A). Next, groups of naïve mice (four to five animals in each of two combined experiments) were inoculated intraperitoneally with the viable cells or with their homogenates, as well as with two dilutions (10^-2 ^and 10^-4^) of scrapie-infected brain homogenates. Each mouse was infected with the equivalent of cells per homogenate harvested from two donor mice, comprising a PrP^Sc ^load comparable to that of a 10^-4 ^dilution of scrapie-infected brains. All groups of infected mice were followed closely for signs of neurological disease. Mild EAE signs, such as limp tail and hind limb weakness (paraparesis), were observed only in the mice inoculated with scrapie-EAE viable cells (Figure 3B), starting at day 30 post infection. Figure 3C presents the survival data for the mice in the different experimental groups. Mice from the groups infected with CFA only-induced cells, as well as with cell homogenates and 10^-4 ^brain dilutions, developed fatal prion disease at about 220 days, indicating no difference in the PrP^Sc ^to infectivity ratio of the homogenates from cells and brain, as well as for viable cells induced only with CFA. Contrarily, the group infected with viable scrapie-EAE cells succumbed to prion disease 30 days earlier, even before the mice infected with a 10^-2 ^scrapie brain dilution. These results indicate that the PrP^Sc ^to infectivity ratio is at least 100 times lower in the activated viable cells, consistent with the possibility that accelerated prion disease is caused by the inflammation-dependent targeting of prions into the CNS.

### PrP^Sc ^in white matter demyelinated areas

**Figure 4:**
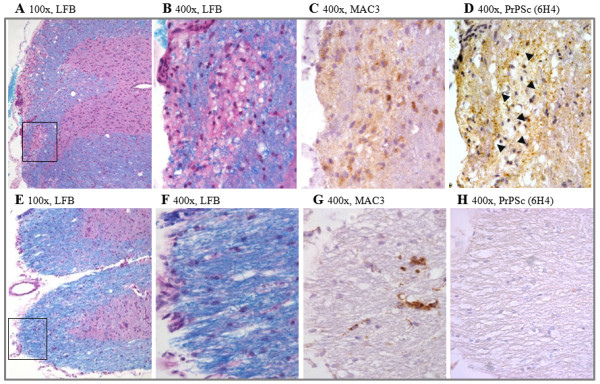
**Pathology spinal cords**. Upper panel: viable cells. Pathology is characterized by marked subpial demyelination (A, B, Luxol fast blue). The rectangle in (A) marks the region enlarged in (B-D). The demyelinated areas show some activated microglia and macrophages (C, Mac-3) as well as moderate PrP^Sc ^deposition, which is most abundant at the lesion border (D, arrowheads, PrP^Sc^). Lower panel: homogenated cells. In contrast, control animals were devoid of demyelination (E, F, luxol fast blue). The rectangle in (E) marks the region enlarged in (F-H). White matter only reveals some activated microglia (G, Mac-3) but no PrP^Sc ^deposition (H).

**Figure 5:**
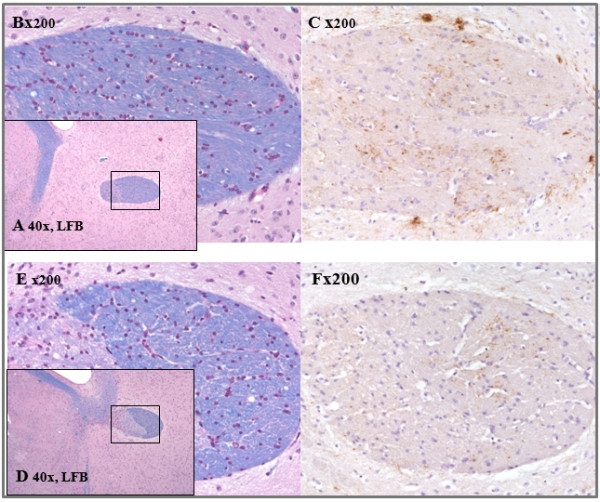
**Pathology of brains**. Upper panel: viable cells. The commissura anterior area of mice infected with viable cells (A, B, Luxol Fast Blue) showed extensive PrP^Sc ^deposition (C, PrP^Sc^) in contrast to animals infected with cell homogenates (D, E, Luxol Fast Blue) that revealed less PrP^Sc ^deposition in this anatomical region (F, PrP^Sc^).

The pathological examination of scrapie-infected mice induced for EAE [20] revealed depositions of PrP^Sc ^in white matter inflamed areas. Figure 4 shows the differences in demyelination and PrP^Sc ^deposition in the white matter spinal cords of naïve mice infected with viable as compared to homogenized scrapie-EAE cells. Indeed, spinal cords of mice infected with viable scrapie-EAE cells present marked subpial demyelination co-localized with activated microglia, macrophages and moderate levels of PrP^Sc ^accumulation, mainly at the border of the lesion (Figure 4). Due to the long time lapse from infection with the viable cells to prion disease manifestation, it is hard to determine whether prion-infected macrophages were the ones that deposited the PrP^Sc ^in the demyelinated spinal cord, or whether their presence represents the engulfing of other prion-infected cells, such as T-cells. Such a co-localization between PrP^Sc^, Mac-3 and demyelination was not present in the spinal cords of mice infected with cells homogenates. Extensive PrP^Sc ^deposition was also observed in brain white matter areas such as the commissura anterior of mice infected with scrapie-EAE viable cells (Figure 5), again contrary to the low levels of PrP^Sc ^observed in the brain white matter of mice infected with cell homogenates. These results are again consistent with the idea that viable prion-infected immune cells activated against myelin components, such as MOG in this case, may carry PrP^Sc ^into white matter CNS areas, a feature not frequently seen in prion infection [21]. For unknown reasons, this route of infection is more virulent, as compared to classic prion neuroinvasion, and is believed to involve retrograde transport of prions to the brain. Both routes of neuroinvasion may occur in parallel and produce additive signs of disease.

### Transmission of disease from scrapie-experimental autoimmune encephalomyelitis inoculated brains

We have shown in Figure 3 that while most of the inoculated samples comprised similar levels of PrP^Sc^, the sample consisting of viable scrapie-EAE cells generated disease in the naïve mice significantly earlier than all others, indicating that levels of PrP^Sc ^are not the only designator of prion disease incubation time. In our previous work [20], we showed that mice may succumb to the co-induced scrapie-EAE disease at similar incubation times with very different levels of brain PrP^Sc^, those varying from undetectable levels to the high levels observed in mice with classical scrapie. To this effect, we next investigated whether mice succumbing to the co-induced disease generate a new prion strain that can transmit disease independent of PrP^Sc ^inoculum levels, or whether disease transmission from these mice correlate with the accumulated PrP^Sc ^levels when in brain homogenates.

**Figure 6:**
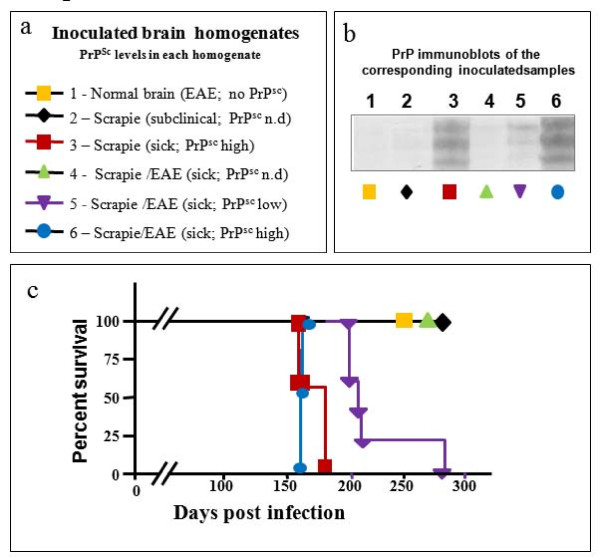
**Secondary transmission of co-induced brain homogenates depends on PrP^Sc ^levels**. **(A) **Sample legend. **(B) **Immunoblots presenting levels of PrP^Sc ^in the samples described in A. **(C) **Survival curves of mice (n = 5 for each group) infected with the samples described in A and B. The survival curve of scrapie-EAE (PrP^Sc ^high) was significantly different (*P *< 0.02) as compared to scrapie-EAE (PrP^Sc ^low).

To separate these possibilities, we infected groups of 5 C57Bl/6 J mice with the brain homogenates of the samples described in Figure 6. Group 1 was inoculated with the brain homogenate from a normal mouse induced for EAE, group 2 was infected with the homogenate of a still healthy mouse incubating classical scrapie, and group 3 was infected with the homogenate of a scrapie sick mouse. Groups 4 to 6 were infected with brains of mice that succumbed to the scrapie-EAE co-induced disease at a similar time point; however, each with a different level of brain PrP^Sc^. Figure 6 shows that samples 1, 2 and 4 could not transmit disease to naïve mice for at least 300 days post infection. This is specifically surprising for sample 4, generated from the brain of a mouse that succumbed to the co-induced disease. Sample 5, which presents marginal levels of PrP^Sc^, generated intermediate levels of infectivity in the recipient group. Only mice inoculated with samples 3 and 6 (high levels of PrP^Sc^) succumbed to prion disease, at short incubation times. These results indicate that the scrapie-EAE syndrome, while causing accelerated disease, does not induce the replication and accumulation of prions that can further transmit disease in the absence of PrP^Sc^. These results are therefore consistent with the notion that inflammation-dependent targeting of PrP^Sc ^can accelerate disease regardless of the inoculated PrP^Sc ^levels. In the absence of such PrP^Sc ^targeting, as is the case for the total brain homogenates in these second transmission experiments, it is the levels of PrP^Sc ^that determine the incubation time of disease manifestation.

## Conclusions

We have shown in this work that acceleration of prion disease by CNS inflammation, as is the case for EAE induction, requires the presence of substantial levels of PrP^Sc ^in the immune cells of prion-infected mice. Such PrP^Sc^-rich cells may then be targeted by CNS inflammation for infiltration into the CNS, as demonstrated by the presence of PrP^Sc ^deposits in white mater areas of brains and spinal cords. This was true for mice induced with EAE a month after scrapie infection [20] as well as for mice infected directly with viable and activated scrapie-EAE cells (Figures 3 and 4). Our experiments also show that the inflammation-mediated disease does not constitute a more virulent prion strain, since secondary transmission from scrapie-EAE mouse homogenized brain samples indicated that prion titers were dependent mostly on the inoculated levels of PrP^Sc^. These results are consistent with previous observations that suggest inflammation may affect the sites of PrP^Sc ^accumulation [16,17] and, in the case of CNS inflammation, accelerate the presentation of fatal disease [20], sometimes in the absence of high levels of PrP^Sc ^in the brains of the affected animals. It is possible that low levels of prions that have infiltrated into white matter areas are more toxic and facilitate the propagation of disease in a more efficient way. Both mechanisms, retrograde transport of prions and inflammation-dependent neuroinvasion, may occur distinctively or in parallel, explaining how similarly sick mice present different levels of brain PrP^Sc ^at the end point of disease. It is also possible that a variable balance between EAE-linked and scrapie symptoms can account for this phenomenon.

The fact that incubation times for second-generation transmission of disease from homogenates of scrapie-EAE brains were independent of the clinical status of donor mice and related only to PrP^Sc ^levels indicates that inflammation did not change the virulence properties of the initial prion strain, but rather accelerated the targeting of the infectious agent to vital areas, resulting in a shorter disease. This may explain why endogenous blood prions carry more infectivity than predicted from their low blood PrP^Sc ^content [4,26,27]. Indeed, our results suggest that infection by viable cells carrying PrP^Sc ^may be more rapid and that the infectivity of a specific blood sample may depend not only on the prion incubation status of the blood donor, but also on the inflammatory state of both the donor and recipient on the day of the transfusion.

## Competing interests

The authors declare that they have no competing interests.

## Authors' contributions

YF-L made substantial contributions to the conception and design, acquisition of data, analysis and interpretation of results and drafting of the manuscript. RH made substantial contributions to the acquisition of data and its interpretation. HB made substantial contributions to the acquisition of data, interpretation of data and critical reading of the manuscript. TM-S made substantial contributions to the acquisition of data and interpretation of results. OA made substantial contributions to the interpretation of data and critical reading of the manuscript. HO made substantial contributions to the conception and design, acquisition of data, and interpretation of results and critical reading of the manuscript. RG made substantial contributions to the conception and design, acquisition of data, interpretation of results, drafting the manuscript, and has given final approval of the version to be published. All authors read an approved the final manuscript.
